# Joint and independent neurotoxic effects of early life exposures to a chemical mixture: A multi-pollutant approach combining ensemble learning and G-computation

**DOI:** 10.1097/EE9.0000000000000063

**Published:** 2019-09-23

**Authors:** Youssef Oulhote, Brent Coull, Marie-Abele Bind, Frodi Debes, Flemming Nielsen, Ibon Tamayo, Pal Weihe, Philippe Grandjean

**Affiliations:** a Department of Biostatistics and Epidemiology, School of Public Health and Health Sciences, UMASS- Amherst, Amherst, Massachusetts; b Department of Environmental Health, Harvard T. H. Chan School of Public Health, Boston, Massachusetts; c Department of Biostatistics, Harvard T. H. Chan School of Public Health, Boston, Massachusetts; d Department of Statistics, Faculty of Arts and Sciences, Harvard University, Cambridge, Massachusetts; e Department of Occupational Medicine and Public Health, Faroese Hospital System, Torshavn, Faroe Islands; f Institute of Public Health, University of Southern Denmark, Odense, Denmark

**Keywords:** Chemical mixtures, Children, G-computation, mercury, Neurodevelopment, Perfluoroalkyl substances, Superlearner

## Abstract

**Background:**

Exposure to mercury (Hg) is associated with adverse developmental effects. However, Hg occurs with a multitude of chemicals. We assessed the associations of developmental exposure to multiple pollutants with children’s neurodevelopment using a novel approach.

**Methods:**

Hg, polychlorinated biphenyls (PCBs), and perfluoroalkyl substances (PFASs) were measured in maternal and children’s blood at 5 years (n = 449 and 419). At 7 years, children were administered Boston Naming Test (BNT) and the Strengths and Difficulties Questionnaire (SDQ). We used the G-formula combined with SuperLearner to estimate independent and joint effects of chemicals at both ages. We constructed flexible exposure-response relationships and assessed interactions.

**Results:**

Most chemicals showed negative relationships with BNT scores. An interquartile range (IQR) increase in maternal Hg and perfluorooctanoic acid (PFOA) was associated with 0.15 standard deviation (SD) (95% confidence interval [CI] = –0.29, –0.03) and 0.14 SD (95% CI = –0.26, –0.05) lower scores in BNT, whereas a joint IQR increase in the mixture of chemicals was associated with 0.48 SD (95% CI = –0.69, –0.25) lower scores in BNT. An IQR increase in PFOA was associated with 0.11 SD (95% CI = 0.02, 0.26) higher total SDQ difficulties scores. Maternal ∑PCBs concentrations were associated with lower SDQ scores (β = –0.09 SD; 95% CI = –0.19, 0), whereas 5 years ∑PCBs showed a negative association (β = –0.09 SD; 95% CI = –0.21, 0). Finally, a joint IQR increase in the mixture was associated with 0.22 SD (95% CI = 0.04, 0.4) higher SDQ scores.

**Conclusions:**

Using a novel statistical approach, we confirmed associations between prenatal mercury exposure and lower cognitive function. The potential developmental effects of PFASs need additional attention.

## Abstract 2

Supplemental Digital Content is available in the text.

**What this study adds**

Strong epidemiologic evidence suggests that early life exposure to certain chemicals impairs brain development, and therefore lead to decreased cognitive function. However, the majority of previous studies used a single pollutant-at-a-time approach, considering each chemical separately when assessing the potential effects of chemicals. In this study, we jointly investigated the potential neurodevelopmental effects of maternal and child 5-year exposure to mercury (Hg), perfluoroalkyl substances (PFASs), and polychlorinated biphenyls (PCBs) using a novel approach that combines the SuperLearner with G-computation. We found evidence that higher maternal Hg and PFOA concentrations were associated with decreases in cognitive function. The joint effect of the mixtures of chemicals was stronger, but no potential for synergistic effects was observed.

## Background

Mercury (Hg) and other persistent organic pollutants (POPs) are environmental chemicals that are ubiquitous, persistent, and accumulate in marine mammals, with well-documented potential for developmental toxicity. Strong epidemiologic evidence suggests that many of these chemicals are neurotoxicants with potential to harm the developing brain, therefore resulting in long-lasting neurodevelopmental sequels.^1,2^ The large majority of environmental epidemiology studies typically adopt a single pollutant-at-a-time approach, considering each chemical separately when assessing the potential neurotoxic effects of chemicals; therefore, providing only limited insights on the real environment and health associations. Indeed, biomonitoring studies of environmental chemicals demonstrate that the general population experiences exposure to multiple chemicals from many different sources and at varying levels. Traditionally, chemical mixtures have been studied via. multivariable parametric regression approaches that mutually adjust for mixture components and estimate the independent effect of each component, while adjusting for the others. Recently, several statistical methods have been proposed to estimate health effects of environmental mixtures, often with an emphasis on variable selection.^3^ These methods include environmental-wide association studies (EWAS),^4^ penalized regression methods (e.g., least angle selection and shrinkage operator [LASSO]),^5^ dimension reduction methods, and exposure-response surface methodology such as generalized additive models (GAMs)and kernel regression methods.^3,6^

In this article, we investigated the potential independent and joint neurodevelopmental effects of maternal and child 5-year exposure to seven major marine pollutants in a population of Faroese children 7 years of age. We propose to use an ensemble machine learning technique called Super Learner^7^ that offers greater flexibility in approximating the data generating mechanism, and we combine it with G-computation,^8,9^ a causal inference approach that can yield valid causal effect estimates. This proposed approach can mitigate the problems of multicollinearity and model misspecification, with nonparametric prediction algorithms fitting complex exposure-response curves (Oulhote et al).^32^ We apply this approach to estimate valid exposure-response relationships and detect potential interactions.

## Methods

### Study population

The birth cohort was formed from 656 consecutive pregnancies recruited at the last antenatal examination at week 32 of pregnancy at the National Hospital in Tórshavn, Faroe Islands, during 1997–2000.^10^ The cohort can be considered reasonably representative of Faroese births. The Faroe Islands are located in the North Atlantic Ocean, between Norway and Iceland. The Faroese population is fairly homogeneous mainly of Scandinavian origin.^11^ Populations in the Faroese have depended on a traditional diet that includes fish, pilot whale, sheep, and birds. Therefore, concentrations of mercury and persistent organic pollutants in Faroese residents have been shown to be elevated compared with other populations especially due to dietary intake of pilot whale.^11,12^ Given the fairly homogenous population in terms of genetics and socioeconomic status (SES), and an exposure to POPs and related contaminants that covers a range of over 100-fold, this population offer a unique opportunity to study health effects of environmental contaminants with a limited potential for confounding.

Of the 656 pregnancies, 640 singleton births were included. Obstetric variables, including date of birth, birth weight, parity, and maternal age, were obtained from obstetrical and medical records. Information on prepregnancy weight and height, socioeconomic status, maternal smoking, and alcohol use during pregnancy were self-reported. The birth cohort underwent a prospective follow-up at age 7 years. A maternal interview informed questions concerning current health and past medical history, lifestyle, duration of breastfeeding, behavior, and other characteristics.

The study protocol was approved by the ethical review committee serving the Faroe Islands and by the institutional review board at the Harvard School of Public Health, and written informed consent was obtained from all mothers.

### Assessment of children’s cognitive and behavioral functions at 7 years of age

For the purpose of this explorative investigation, we restricted the outcomes’ assessment to two neuropsychological tools are as follows: (1) the Boston Naming Test (BNT) that has been shown to be particularly sensitive to methylmercury exposure and 2) the Strengths and Difficulties Questionnaire (SDQ) for which we previously reported associations with environmental exposures in single pollutant approaches.^13–17^ A total of 567 children underwent neuropsychological assessment at age 7 years.

### Boston Naming Test

The 60-item BNT^18^ is a visual confrontation naming test which measures the word retrieval or word finding performance of a subject. Stimuli are line drawings of a wide category of objects of increasing difficulty. Scores are obtained for number of correct items without cueing, and correct number of items after stimulus and phonemic cueing by the examiner.^17^

### Strengths and Difficulties Questionnaire

The Parent’s version of the SDQ^19^ is comprised of 25 items scored on a 3-point Likert scale. Five behavioral subscales with a score range of 0 to 10 are calculated from the 25 SDQ items: emotional symptoms, conduct problems, hyperactivity/inattention, peer relationship problems, and prosocial behavior. A total difficulties score ranging from 0 to 40 was calculated by summing four of the subscales (emotional, conduct, hyperactivity, and peer). Higher total SDQ scores indicate higher behavioral difficulties.

### Exposure assessment

Maternal exposures were assessed at the last antenatal examination at week 32 of pregnancy; 5-year exposure was assessed from the child blood at age 5.

Total mercury (Hg) concentration in whole blood was determined on a Direct Mercury Analyzer (DMA-80; Milestone Inc, Sorrisole, Italy).^20^ Serum polychlorinated biphenyls (PCBs) concentrations were measured after solid-phase extraction (SPE) using gas chromatography equipped with an electron capture detector (μ-ECD).^21^ To avoid problems with congeners not assessed and concentrations below the detection limit, a simplified ΣPCB concentration was calculated as the sum of major congeners CB-138, CB-153, and CB-180 multiplied by 2.^22^ The concentrations of PCBs are expressed in relation to the total lipid concentration determined using the Cypress Diagnostics kit (Langdorp, Belgium). Perfluorooctanoic acid (PFOA), perfluorooctane sulfonic acid (PFOS), perfluorohexane sulfonic acid (PFHxS), perfluorononanoic acid (PFNA), and perfluorodecanoic acid (PFDA) concentrations were measured using online solid-phase extraction and analyzed using high-pressure liquid chromatography with tandem mass spectrometry.^23^ The analysis of mercury, PCB’s, and PFAS’s were all conducted at Department of Environmental Medicine, Institute of Public Health, University of Southern Denmark.

In children with neuropsychological test scores at age 7 years, complete measurements of chemicals concentrations were available for 465 and 503 children respectively for maternal and 5-year exposures. Additionally, complete measurements for both maternal and 5-year exposures were available for 430 children with neuropsychological test scores (Supplemental Material; Figure S1; http://links.lww.com/EE/A53).

### Covariates and potential confounders

We collected sociodemographic and lifestyle factors and medical history during pregnancy and at delivery via. administered questionnaires. At the 5-year follow-up visit, duration of exclusive breastfeeding was reported. We considered the following potential covariates in our models: child’s exact age (in months), sex (boy; girl), birth weight (grams), maternal age at pregnancy, prepregnancy body mass index (body mass index [BMI], kg/m^2^), parity (nulliparous; primipara; and multiparous), duration of exclusive breastfeeding (months), maternal intelligence (RAVEN scores), maternal socioeconomic status (SES) during pregnancy based on education (low: ≤10 years of education; intermediate: school leaving certificate and above including technical studies; and high: university studies), alcohol consumption during pregnancy (never; ever), and smoking during pregnancy (no; 1–5 cigarettes/day; more than 5 cigarettes/day). Final models for prenatal pollutant exposures included age, sex, maternal age, prepregnancy BMI, parity, maternal RAVEN scores, socioeconomic status, and alcohol and smoking during pregnancy. Models for child 5-year exposures further included prenatal pollutant exposures, duration of exclusive breastfeeding and birth weight.

### Statistical analyses

Complete data on exposures, outcomes, and covariates were available for 449 and 419 children, respectively, for maternal and 5-year exposures (See supplemental material; Figure S1; http://links.lww.com/EE/A53). We did not conduct any imputations as the methods presented in this article could not be conducted on multiply imputed datasets. Mercury, PFASs, and ∑PCBs concentrations were logarithmically (base 10) transformed and centered. Initial exploratory data analyses included descriptive statistics and univariate associations between exposures and outcomes and potential covariates of interest. Neuropsychological outcomes were standardized with a mean of 0 and standard deviation (SD) of 1 in analyses investigating associations between multiple contaminants and neuropsychological functions. Estimates therefore represent the SD change in the outcome related to an interquartile range (IQR) increase in exposures.

To estimate individual and joint estimates of the associations between environmental exposures and neuropsychological scores, we first generated a valid prediction of the outcomes using the SuperLearner algorithm. SuperLearner is a data-adaptive approach that has been proposed by van der Laan et al.^7,24,25^ It uses cross-validated risks to find an optimal combination of predictions from a list of algorithms supplied by the user that minimizes a given loss function (e.g., squared error). We included a set of prediction algorithms in the library that can cover a large range of exposure-response relationships: the generalized linear regression model (GLM) and generalized additive models (GAM), elastic net regularization,^26^ multivariate adaptive polynomial spline regression,^27^ support vector machine,^28^ gradient boosting,^29^ random forests,^30^ and artificial neural networks.^31,32^

After obtaining a valid model for the outcome given the concurrent exposures and other covariates using SuperLearner, the resulting model was used to predict the neuropsychological test scores under specified exposure scenarios using G-computation.^8,9^ The marginal effect (hereafter called naïve average causal effect [NACE]) is estimated by calculating the sample average of the model predictions if exposure is set to the 75th percentile of exposure for all individuals minus the sample average of the model predictions if exposure is set to the 25th percentile of exposure for all individuals, while leaving the values of the remaining covariates at their observed values. For instance, the NACE for an IQR increase in maternal blood Hg is expressed as follows: 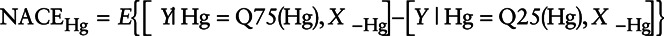, where Q75 and Q25 are respectively the 75th and 25th percentiles of Hg distribution and 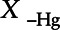 includes all the remaining covariates (including exposures and confounders) required for the identifiability of the effect estimate. The same strategy was applied to investigate the joint effect of the chemical mixture by replacing all exposures of interest first by the 75th percentile for all individuals in the cohort and then by the 25th percentile in a second time. In both cases, predictions of the outcome were calculated for all individuals, and we reported the difference between the sample averages of both sets of predictions.

In the absence of a theoretical formula for the asymptotic distributions of these parameters within the SuperLearner, we used bootstrapping (N = 200) to approximate the 95% confidence intervals (CIs).^33,34^

Since the NACE cannot reveal nonlinearities, we therefore constructed dose-response relationships for each exposure. We used SuperLearner to predict test scores when replacing exposure values by a specific percentile of that exposure for all subjects while keeping the values of the other exposures and covariates at their observed levels. We calculated the sample average of those predictions to obtain the predicted response at a given exposure percentile, and the process was repeated for several exposure percentiles. The resulting values were used to plot a curve of the partial relationship between Y and X_*j*_, which we called 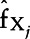. The average partial relationship between X_*j*_ and Y can be therefore expressed as follows: 
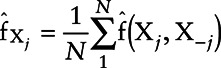
, where X_*j*_ is the exposure of interest, 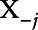 is the remaining set of exposures and covariates, N is the number of observations and 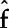 denotes predictions from the SuperLearner.

Finally, to investigate the presence of potential interactions between exposures or effect modification by other covariates, we used individual conditional expectations (ICEs) for each exposure.^35^ The ICE was calculated for each individual in the dataset separately for each exposure. For a given exposure, the ICE of a subject was calculated by replacing the value of that exposure by a given exposure percentile and computing the model predictions while keeping the values of the other exposures and covariates at his/her observed levels. Repeating the process for several exposure percentiles, we obtained the curve for that individual and exposure. The process was then repeated for all exposures and individuals. We therefore plotted N estimated conditional expectation curves, each reflecting the individual predicted response as a function of the exposure X_*j*_, conditional on the observed 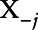.^35^

We did not perform any null hypothesis significance testing since we are not making any decisions. Rather, we rely on interval estimation for statistical inference and present the resulting estimates for all the exposures with their 95% CI based on nonparametric 2.5% and 97.5% percentiles.

## Results

Mean age at examination was 89.9 months (IQR: 89–91), and there was a comparable number of boys and girls (49% and 51%, respectively). Most of the children had an older sibling (75%), and 32% were exclusively breastfed for 6 months or longer (Supplemental Material: Table S1; http://links.lww.com/EE/A53). Maternal age at delivery was 29.5 years (IQR: 26–33), and 8% of mothers reported a prepregnancy BMI higher than 30. Twenty-seven percent of the mothers reported ever smoking during pregnancy, and 41% reported ever consuming alcohol during pregnancy. Finally, mean maternal Raven intelligence score was 48.6 (IQR: 45–53). The 449 and 419 children included respectively in the prenatal and 5-year analyses did not significantly differ from the overall children included in the neuropsychological assessment at 7 years in terms of important characteristics (Supplemental Material; Table S1; http://links.lww.com/EE/A53).

Table 1 describes univariate associations between neuropsychological endpoints and important characteristics of the study population for the 449 included children. Mean score for BNT without cues (number of correct items without cueing) was 27.4 and mean BNT with cues (number of correct items after stimulus and phonemic cueing) was 30.3. Mean SDQ total difficulties was 6.4. BNT scores with and without cues were higher among children with no older siblings, of mothers with high SES and high Raven scores, and who reported ever drinking during pregnancy. Total SDQ difficulties scores were higher (indicating more problems) in children of younger mothers, with low SES, and who reported ever smoking during pregnancy.

**Table 1:**
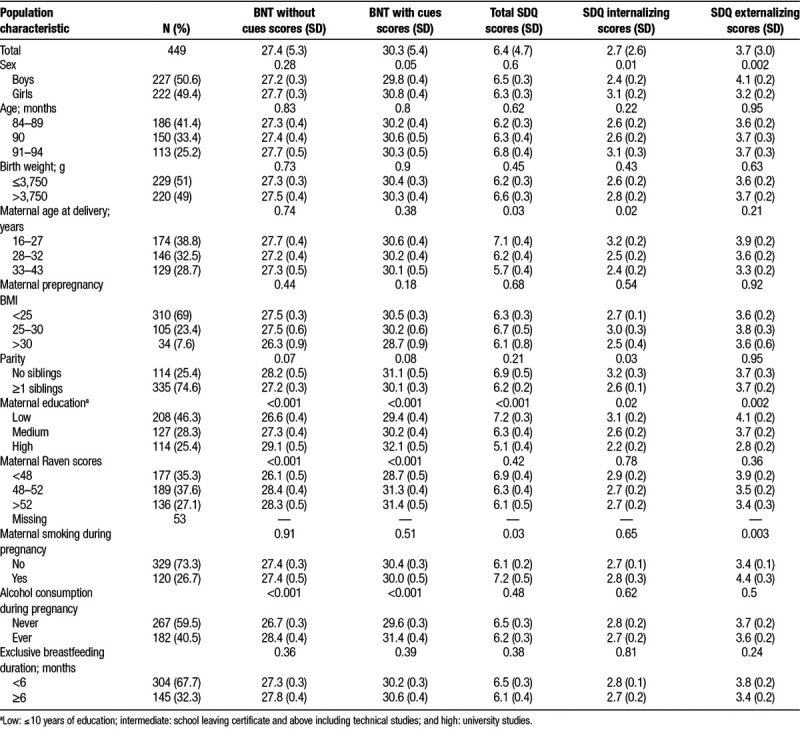
Univariate associations between neuropsychological endpoints and important characteristics of the study population included in the prenatal analyses

Table 2 shows distributions of maternal and 5-year environmental exposures. Among PFASs, PFOS showed the highest serum concentrations at all timepoints, followed by PFOA and PFHxS. Maternal PFOS and PFHxS concentrations were higher in comparison to 5-year child concentrations, whereas PFOA, PFNA, and PFDA concentrations were comparable between maternal and children at 5 years. Blood Hg concentrations were also higher in maternal blood compared with children’s blood, whereas ∑PCB concentrations were comparable.

**Table 2:**
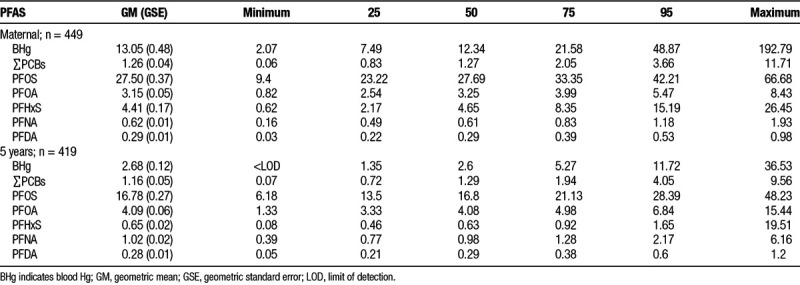
Descriptive statistics of maternal and child’s 5-year pollutants concentrations measured in blood

**Figure 1.**
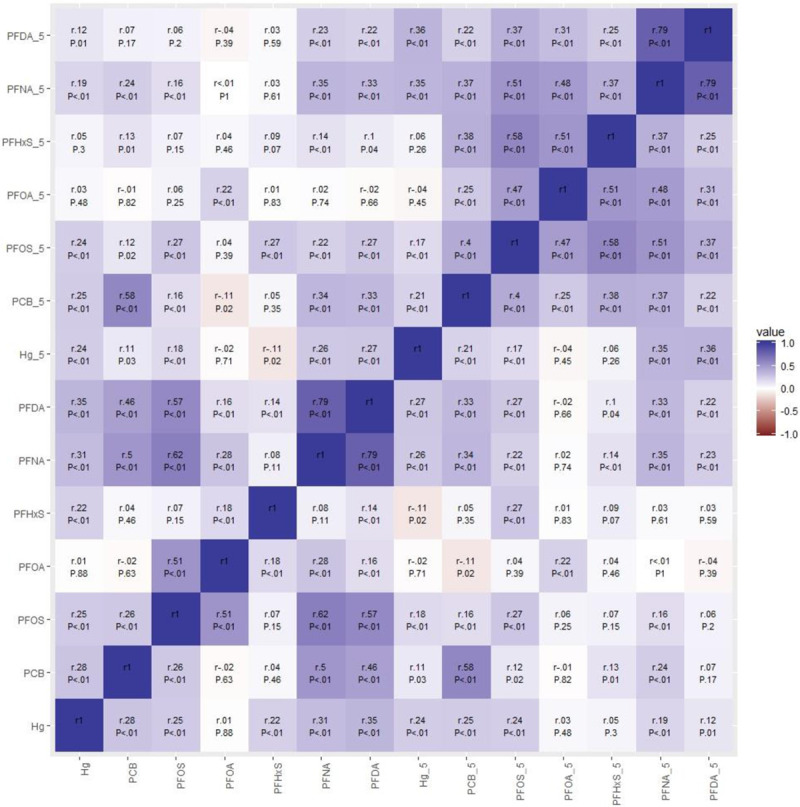
Correlation plot of prenatal and 5-year concentrations. Red indicates negative correlations, whereas blue indicates positive correlations. The intensity of the color indicates the strength of the correlation.

Figure 1 presents a heat map plot of within (at the same time point) and between (exposure between maternal and 5 years) exposures correlations. The highest within correlations (Spearman’s *ρ*) were observed between serum concentrations of PFDA and PFNA (*ρ* = 0.79 at both maternal and 5 years), whereas the lowest correlations were observed between serum PFOA and Hg concentrations at 5 years (*ρ* = –0.04). The highest between-correlation was observed between maternal and 5 years serum ∑PCB concentrations (*ρ* = 0.58), whereas the lowest correlation was observed between maternal and 5 years serum PFHxS concentrations (*ρ* = 0.09).

### Univariate associations

Maternal Hg, PFOS, and 5 years PFHxS concentrations were negatively associated with BNT scores. Maternal PFOS concentrations were also associated with higher (poorer) total SDQ scores, whereas 5-year ∑PCB concentrations were associated with lower total SDQ scores (Supplemental Material: Figure S1; http://links.lww.com/EE/A53).

### Insights from SuperLearner cross-validation

**Figure 2.**
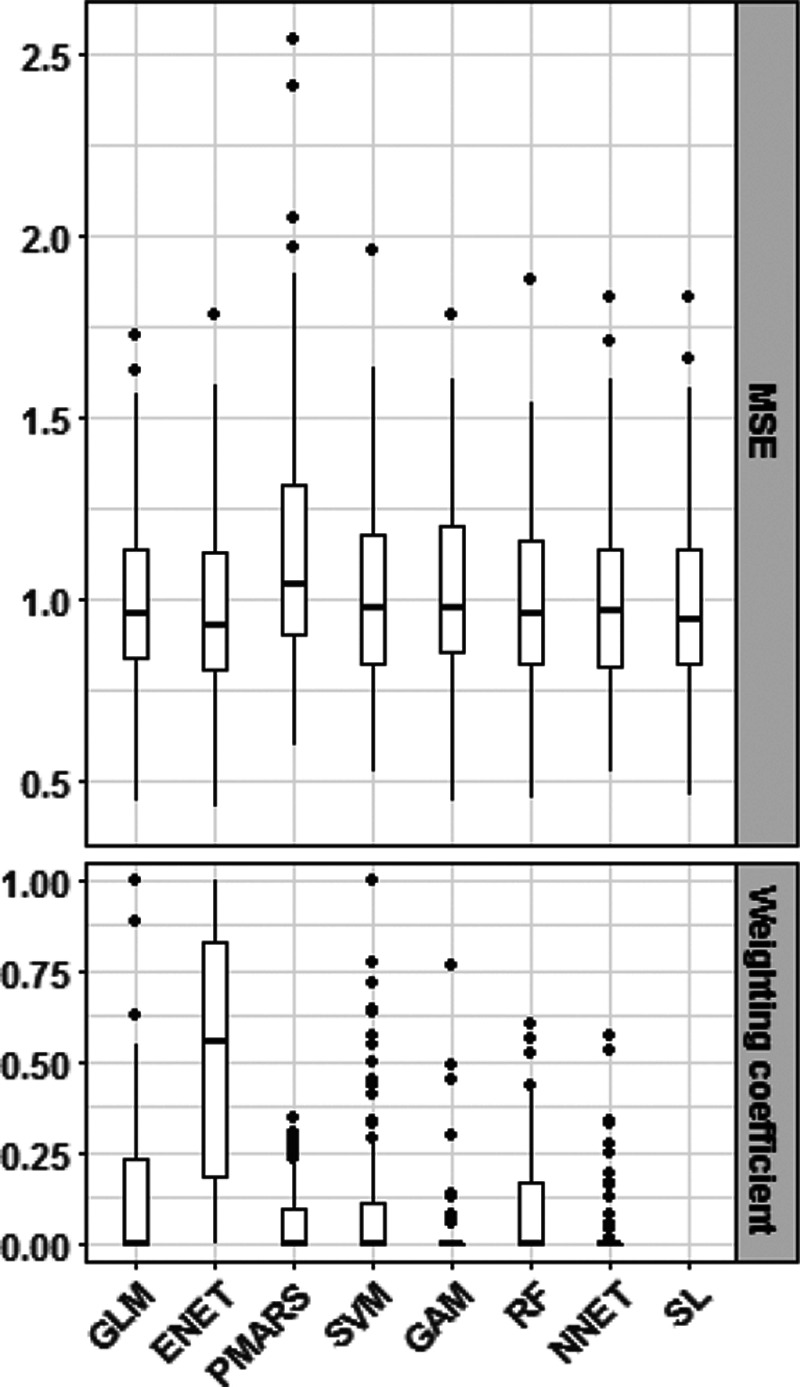
Distribution of the 10-fold cross-validated minimum squared error and weighting coefficients for each included algorithm and for the SuperLearner. ENET indicates elastic net regularization; NNET, artificial neural networks; PMARS, multivariate adaptive polynomial spline regression; RF, random forests; SVM, support vector machine and SL, Super learner.

Figure 2 shows the distribution of the 10-fold cross-validated minimum squared error (MSE) for each included algorithm and for the SuperLearner. The figure also shows the distribution of the weighting coefficients of the convex combination applied to minimize the prediction error for each algorithm. The SuperLearner and the Elastic net algorithms yielded the lowest average MSE, although the differences were small with the other methods. However, Elastic net highly contributed to the convex combination that yielded the best predictions with a median weighting coefficient of 0.56. All the remaining algorithms included in the SuperLearner had a median weighting coefficient of 0.

The Elastic net algorithm outperformed all the other included algorithms, which points to two main insights: (1) the exposure-response relationships are likely linear and (2) the absence of potential interactive effects between exposures and between exposures and potential confounders.

### Associations between chemical exposures and neuropsychological outcomes

Table 3 shows the estimates resulting from the G-computation method combined with SuperLearner predictions. An IQR increase in maternal Hg concentrations was associated with 0.08 SD (95% CI = –0.18, 0) and 0.15 SD (95% CI = –0.29, –0.03) lower scores in the BNT without and with cues, respectively. Maternal PFOA concentrations were also associated with lower BNT scores (β = 0.07 SD; 95% CI = –0.16, 0 and 0.14 SD; 95% CI = –0.26, –0.05 for BNT without and with cues, respectively). Finally, an IQR increase in maternal PFOS concentrations was associated with 0.11 SD (95% CI = –0.27, 0.01) lower BNT with cues scores (Table 3). A joint IQR increase in the mixture concentrations was associated with 0.15 SD (95% CI, –0.41, 0.13) and 0.48 SD (95% CI = –0.69, –0.25) lower scores in the BNT without and with cues. Regarding SDQ scores, an IQR increase in maternal PFOS and PFOA concentrations were associated with 0.15 SD (95% CI = 0.08, 0.23) and 0.11 SD (95% CI = 0.02, 0.26) higher total SDQ scores, whereas an IQR increase in maternal ∑PCBs concentrations was associated with 0.09 SD (95% CI = –0.19, 0) lower SDQ scores. A joint IQR increase in the mixture concentrations was associated with 0.19 SD (95% CI = –0.11, 0.42) higher total SDQ scores. No other pattern of associations was observed.

**Table 3:**
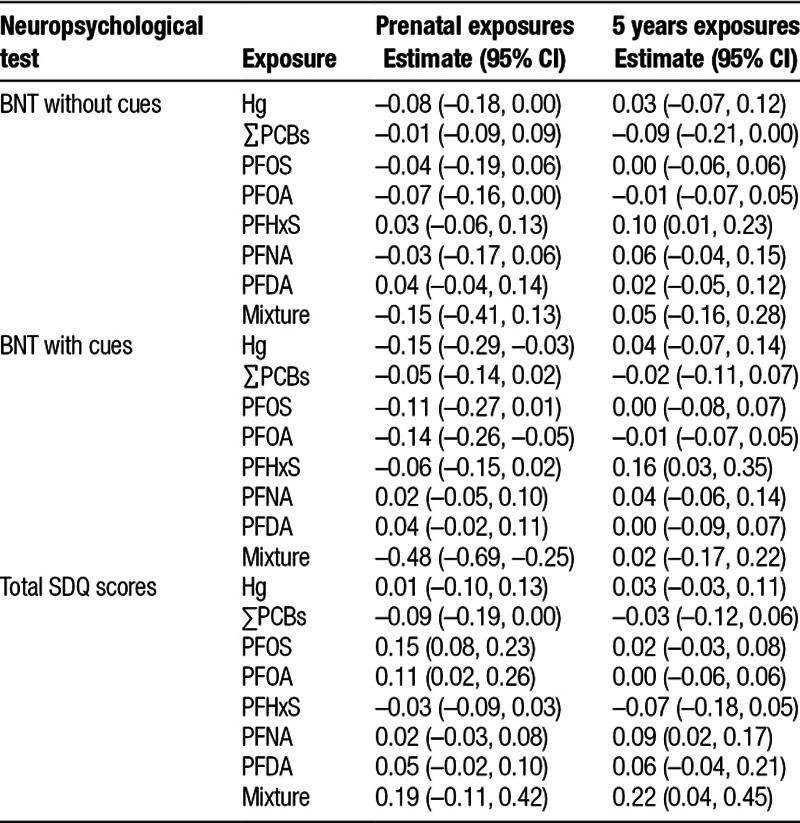
Associations between prenatal and 5 years exposures and neuropsychological test scores at 7 years using G-computation and SuperLearner predictions

Regarding child 5-year exposures, we observed both positive and negative associations with BNT scores. An IQR increase in 5 years ∑PCB concentrations was associated with 0.09 SD (95% CI = –0.21, 0) lower BNT with cues scores, whereas an IQR increase in 5 years PFHxS concentrations was associated with 0.16 SD (95% CI = 0.03, 0.35) higher BNT with cues scores (Table 3). No association was observed for a joint increase in the chemical mixture. Regarding SDQ scores, an IQR increase in 5-year PFNA concentrations was associated with 0.09 SD (95% CI = 0.02, 0.17) higher total SDQ scores. Finally, a joint IQR increase in the mixture of chemicals was associated with 0.22 SD (95% CI = 0.04, 0.45) higher total SDQ scores.

**Figure 3.**
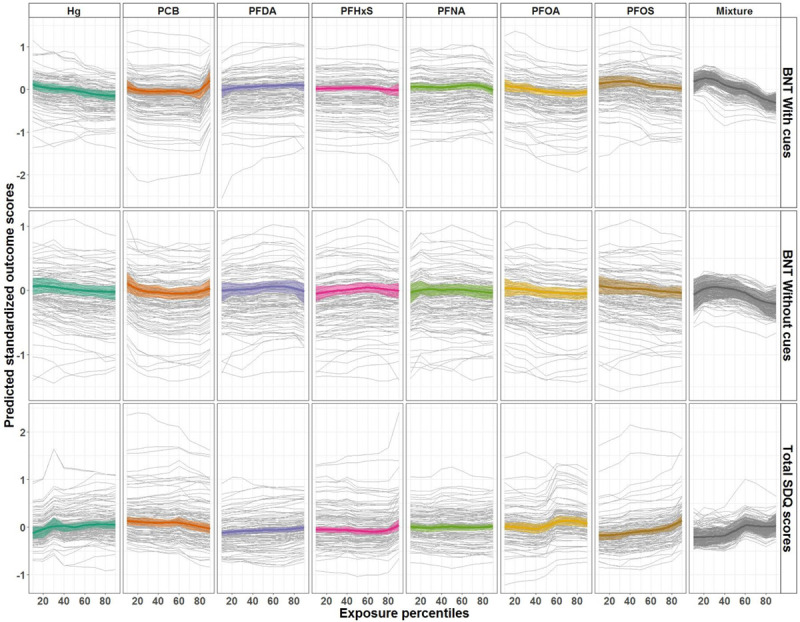
Exposure-response relationships and individual conditional expectations for prenatal exposures and neuropsychological test scores at 7 years using G-computation and SuperLearner predictions. The lines represent locally weighted scatterplot smoothing for mean predictions at different percentiles. For the sake of clarity, only 100 individuals were randomly sampled to represent the individual conditional expectations.

Figure 3 shows the individual conditional expectations and exposure-response relationships for maternal exposure to individual chemicals and the mixture of chemicals. Most observed associations showed a linear pattern, however, some associations showed patterns of nonlinear relationships. The association between ∑PCBs and BNT with cues showed a slight increase after the 80th percentile, whereas the association for the joint effect of chemicals with BNT scores showed a steeper decrease after the 30th percentile for BNT with cues and after the 60th percentile for BNT without cues. Predictions for individuals showed mainly parallel patterns, which points to a lack of potential interactions with other exposures or covariates in the models. At age 5 years, exposure-response relationships exhibited a linear pattern (data not shown).

## Discussion

In this study, we introduce a novel approach combining SuperLearner and G-computation to estimate the associations between both prenatal and child 5-year environmental exposures and child cognitive and behavioral functions at 7 years of age. By investigating all chemicals jointly with no a priori assumptions on the model specification, we were able to account for three main challenges that hamper investigations using single pollutant approaches: (1) multiple comparisons, (2) potential model misspecifications (i.e., nonlinear terms and interactions), and (3) confounding by omitted correlated exposures. Overall, our findings suggest that maternal Hg exposure may be a risk factor of poorer cognitive function, as assessed by the BNT scores. Additionally, PFOA and PFOS also showed negative associations with BNT scores independently of Hg. We also found indications of positive associations between maternal PFOS and PFOA and behavioral problems as assessed by the SDQ. Exposures at age 5 years did not appear to negatively impact child cognitive function at 7 years, but we rather report a positive association between 5 years PFHxS concentrations and BNT scores. Only 5 years PFNA concentrations appeared to be associated with higher SDQ behavioral difficulties scores. The cumulative impact of all the exposures appeared stronger and pointed to a cumulative effect on both cognitive and behavioral functions. We found no evidence supporting potential interactive effects between chemical exposures and no strong indications of nonlinear exposure-response relationships, except for the model for cumulative effects.

Generally, the results from this investigation corroborate our previous findings from individual chemical analyses.^15,16,36^ The chemicals associated with a decrease in cognitive function and those associated with an increase in behavioral problems by individual chemical analyses were also selected as potential risk factors by this novel approach. These include maternal Hg and 5-year PFNA exposures as potential developmental neurotoxicants. Unlike our previous investigation, which also pointed to a positive association between PFOA and PFDA concentrations and higher SDQ behavioral difficulties scores, this joint analysis did not provide evidence of such associations and pointed only to PFNA as a potential culprit, although PFDA also exhibited a pattern of detrimental impact. Instead, maternal PFOA and PFOS concentrations appeared to be associated with lower cognitive function. Although some results suggest a negative slope for ∑PCBs with SDQ behavioral difficulties scores, it appears unlikely that these chemicals may have any positive effects on behavioral function, and this finding may be driven by a correlation between ∑PCBs and unmeasured omega-3 polyunsaturated fatty acids that have been shown to exert a positive effect on cognitive function.^37,38^ Such correlation was not found for mercury and PFASs in a subset of children from this cohort (data not shown). The positive association between 5 years PFHxS concentrations and cognitive function warrants further investigation to elucidate whether this might be a false positive or indeed a true beneficial effect. Some previous investigations reported positive associations between some PFASs and neurodevelopment (Stein et al)^39^ and adult memory functions (Gallo et al).^40^ Although unlikely, these favorable associations were hypothesized to be mediated by the activation of the peroxisome proliferator-activated receptor (PPAR) γ receptor that has been shown to prevent the expression of inflammatory cytokines and other inflammatory mediators in brains of Alzheimer disease animal models.^41^ Also, some PPAR agonist drugs have been proposed as preventive drugs for neurodegenerative conditions, including Alzheimer dementia.^42^ However, the question of why would only PFHxS have these favorable effects while other PFASs showed negative associations with cognitive function remains to be settled. It is difficult to compare doses between the two contexts of an environmental exposure such as PFAS and a therapeutic drug. However, drugs impacting PPAR have shown decreased levels of amyloid-β peptide and the number of activated microglia and astrocytes only in the context of high doses as lower doses showed only modest effects on plaque burden or microglia activation. Thus, our interpretation of this potential positive effect remains speculative.^42^

The findings from the present study point to weak associations for independent exposures, with the strongest estimate being a change of 0.2 SD for an IQR increase. It is therefore worth mentioning that the observed effect sizes in this study are relatively modest, failing to reach the level of clinical significance. However, the estimates regarding the joint effect of the mixture of exposures point to moderate effects that can have a big influence on the prevalence of neurodevelopmental disorders at the population level since subtle effects of chemical exposures may shift the distribution of cognitive and behavioral traits to increase the risk of clinical neurodevelopmental disorders.^12,43^ The impact of a factor at the population level depends not only on the magnitude of its impact on health, or its effect size but also on the distribution of the factor. Given the widespread and ubiquitous exposure to PFAS, these small effect sizes may have a considerable impact at the population level.^44^

A strength of our statistical approach is that it brings together the strong and unparalleled predictive performance the SuperLearner and the G-computation method to open the black box of machine learning techniques. It therefore allows to investigate both the overall potential effect of a mixture and to provide marginal estimates for each exposure, and estimation of dose-response relationships. Under assumptions of conditional exchangeability, consistency, and positivity, these estimates may be interpreted causally. The literature on the use of ensemble learning methods for estimating causal effects is limited,^45^ especially in the field of environmental epidemiology. To our knowledge, this approach has not been explored in settings involving multi-pollutant exposures. A comparable approach is the Bayesian Kernel Machine Regression^3,6^ that uses a kernel regression to estimate the joint exposure-response function of a chemical mixture. One major difference between the two methods is that our approach does not require fixing the levels of other chemicals in the mixture to a specific value when estimating their individual contributions. This allows inferring marginal estimates, and to assess potential interactions visually when the exposure-response relationship varies across individuals.

Other approaches that have been suggested to address the mixtures issue include LASSO,^46^ EWAS,^4,47^ weighted quantile sum regression,^48,49^ and Elastic Net.^50,51^ A major disadvantage of such approaches is that they typically assume specific and often restrictive parametric functional forms for the exposure-response relationship, often resulting in a model that does not accurately capture the complexity of the relationships among high dimensional covariates and health outcomes. Some of these methods, such as penalized regressions result in highly biased estimates as they rely on a procedure that reduces the variance of estimators by introducing substantial bias.^5^

The present work has several limitations. First, the ability of the SuperLearner approach depends on the choice of candidate learners that should be guided by theoretical and practical considerations. We believe that we have included a diverse set of algorithms that can capture a variety of potential exposure-response relationships in addition to interactive effects, if present. Second, we used the bootstrap to estimate valid confidence intervals in the absence of a theoretical formula for the asymptotic distributions of the parameters of interest. This gave rise to a heavy computational burden, especially that the method is based on cross-validation. Further efforts to incorporate the method within a parallel computing framework will substantially reduce the running time. Third, our approach, at this point, does not handle exposure misclassification that can arise from measurement errors, one of the most important issues in environmental epidemiology studies. Finally, and although this was out of the scope of this investigation, our estimates are still based on the overall performance of the predictive model, and require a targeting step to infer doubly robust estimates, which will be incorporated in additional developments.

Regarding the neuropsychological instruments used in this study, we relied on parent-reported SDQ and the BNT for measurement of the outcomes rather than clinical diagnoses. Although there are no studies that validated these tests in the Faroese population, the two tests are part of a battery of tests that was designed to allow assessment of mercury and other potential neurotoxicants associations with deficits in a wide range of abilities and have been previously used in this population.^13,15,16,36^ The SDQ has excellent psychometric properties and was used as a screening and/or assessment tool by psychologists and clinicians.^52–55^ The SDQ has been used to assess children’s behavior across age and culture and is commonly used in longitudinal birth cohorts and national surveys.^56–58^ A recent study confirmed the usefulness of the SDQ as a screening tool for boys and girls across age groups and raters in the general Danish population.^59^

## Conclusions

Our findings from this study point to the neurodevelopmental effect of mercury and corroborate previous results from our Faroese cohort studies using a mixtures approach. Additionally, some PFASs showed a detrimental impact on both cognitive and behavioral functions and deserve more attention in future investigations.

## Conflicts of interest statement

P.G. served as a health expert for the State of Minnesota in a recent lawsuit against a PFAS-producing company. The other authors have no conflicts to report.

Supported by the National Science Foundation-National Institutes of Health’ Oceans and Human Health Program (OCE-1321612).

## Supplementary Material
