# Supplementary material for: Joint and independent neurotoxic effects of early life exposures to a chemical mixture: A multi-pollutant approach combining ensemble learning and G-computation
Source: Environ Epidemiol. 2019 Sep 23;3(5):e063. doi: 10.1097/EE9.0000000000000063 (PMC7015154; doi:10.1097/EE9.0000000000000063)

**Supplementary Material: Joint and independent neurotoxic effects of early life exposures to a chemical mixture: A multi-pollutant approach combining ensemble learning and g-computation**

Youssef Oulhote^1,2^, Brent Coull^2,3^, Marie-Abele Bind ^4^, Frodi Debes^5^, Flemming Nielsen,^6^ Ibon Tamayo^4^, Pal Weihe^5^, Philippe Grandjean^2,6^

1. Department of Biostatistics and Epidemiology, School of Public Health and Health Sciences, UMASS- Amherst, Amherst, MA, USA.
2. Department of Environmental Health, Harvard T. H. Chan School of Public Health, Boston, MA, USA.
3. Department of Biostatistics, Harvard T. H. Chan School of Public Health, Boston, MA, USA.
4. Department of Statistics, Faculty of Arts and Sciences, Harvard University, Cambridge, MA, USA.
5. Department of Occupational Medicine and Public Health, Faroese Hospital System, Torshavn, Faroe Islands.
6. Institute of Public Health, University of Southern Denmark, Odense, Denmark

**Figure S1: Flow diagram of included participants for prenatal and 5-year exposures in relation to neuropsychological test scores.**

**
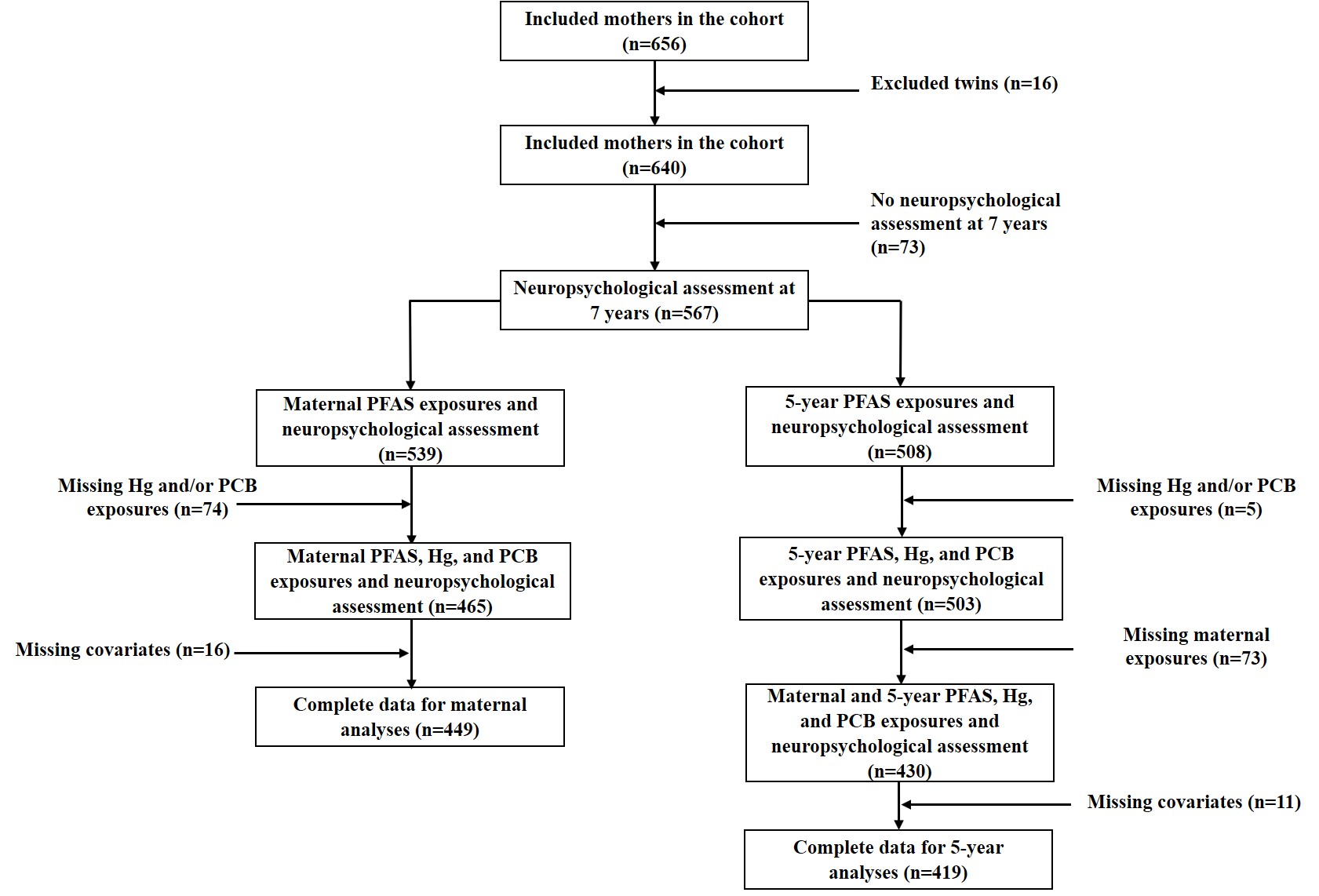
**

**Table S1: Description of the study population**

| **Mean (SD) or n (%)** | | |  |  |  |
| --- | --- | --- | --- | --- | --- |
|  |  |  | **All children included at 7 years follow-up (n=567)** | **Children included in prenatal analyses (n=449)** | **Children included in 5-year analyses (n=419)** |
| *Child characteristics* |  |  |  |  |  |
| Age, months | 89.8 (1.2) | 89.7 (1.1) | 89.7 (1.1) |  |  |
| Birth weight, grams | 3721 (503) | 3724 (497) | 3729 (498) |  |  |
| Sex |  |  |  |  |  |
| Boys | 300 (53 %) | 227 (51 %) | 217 (52%) |  |  |
| Girls | 267 (47 %) | 222 (49 %) | 202 (48%) |  |  |
| *Materal characteristics* |  |  |  |  |  |
| Maternal age at delivery, years | 29.3 (5.1) | 29.5 (5.1) | 29.4 (5.0) |  |  |
| Maternal pre-pregnancy BMI | 23.9 (4.0) | 23.9 (3.8) | 23.8 (3.7) |  |  |
| Parity |  |  |  |  |  |
| No siblings | 154 (27 %) | 114 (25 %) | 107 (25 %) |  |  |
| ≥ 1 siblings | 413 (73 %) | 335 (75 %) | 312 (75 %) |  |  |
| Maternal SES |  |  |  |  |  |
| Low | 272 (48 %) | 208 (46 %) | 189 (45 %) |  |  |
| Medium | 157 (28 %) | 127 (28 %) | 116 (28 %) |  |  |
| High | 138 (24 %) | 114 (26 %) | 114 (27 %) |  |  |
| Maternal Intelligence (RAVEN) | 48.6 (6.0) | 48.6 (6.0) | 48.7 (5.8) |  |  |
| Maternal smoking during pregnancy |  |  |  |  |  |
| No | 417 (74 %) | 329 (73 %) | 311 (74 %) |  |  |
| Yes | 150 (26 %) | 120 (27 %) | 108 (26 %) |  |  |
| Alcohol consumption during pregnancy |  |  |  |  |  |
| Never | 329 (58 %) | 267 (59 %) | 246 (59 %) |  |  |
| Ever | 237 (42 %) | 182 (41 %) | 173 (41%) |  |  |
| Missing | 1 | - | - |  |  |
| Exclusive breastfeeding duration |  |  |  |  |  |
| < 6 months | 364 (68 %) | 304 (68 %) | 278 (66 %) |  |  |
| ≥6 months | 171 (32 %) | 145 (32 %) | 141 (34 %) |  |  |
| Missing | 32 | - | - |  |  |
| *Neuropsychological test scores* |  |  |  |  |  |
| SDQ scores |  |  |  |  |  |
| Total difficulties | 6.6 (4.9) | 6.4 (4.7) | 6.4 (4.6) |  |  |
| Internalizing problems | 2.8 (2.7) | 2.7 (2.6) | 2.7 (2.6) |  |  |
| Externalizing problems | 3.8 (3.2) | 3.7 (3.0) | 3.7 (3.0) |  |  |
| BNT scores |  |  |  |  |  |
| BNT without cues | 27.5 (5.4) | 27.4 (5.3) | 27.5 (5.3) |  |  |
| BNT with cues | 30.4 (5.7) | 30.3 (5.4) | 30.4 (5.4) |  |  |

**Figure S1: Crude unadjusted associations (95% Confidence intervals) between environmental exposures and behavioral and cognitive outcomes**


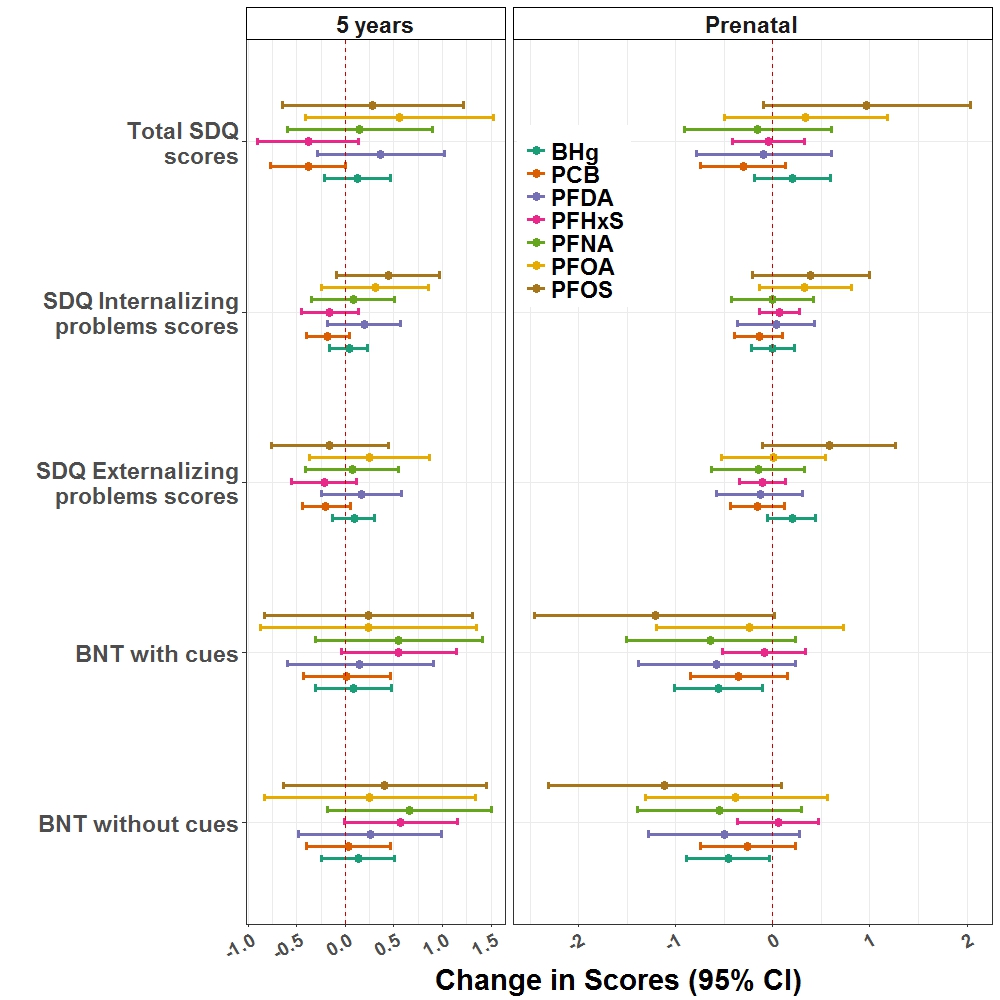

Supplement: Supplementary file 1 [file ee9-3-e063-s001.docx]
